# Resurrection of *Dryotomicus* Wood and description of two new species from the Amazon River Basin (Coleoptera, Curculionidae, Scolytinae, Phloeotribini)

**DOI:** 10.3897/zookeys.56.518

**Published:** 2010-09-17

**Authors:** Anthony I. Cognato, Sarah M. Smith

**Affiliations:** Department of Entomology, Michigan State University, 243 Natural Science, East Lansing, MI 48824

**Keywords:** Scolytidae, bark beetle, taxonomy, tropical biodiversity

## Abstract

A cladistic analysis based on 20 morphological characters was conducted for 11 species representing two valid and two synonymized Phloeotribini genera. One hundred-eighty most-parsimonious trees were recovered and the Dryotomicus Wood species were monophyletic in a mostly unresolved strict-consensus tree. The unusual antennal morphology, with the length of the first two funicular segments equal to the last three segments and a scape which is twice the length of the funicle, distinguish Dryotomicus from the other Phloeotribini genera. Hence this genus is resurrected because of monophyly and diagnostic characters. Dryotomicus oenophilis **sp. n.** and Dryotomicus woodrex **sp. n.** are described from Guyana and Peru, respectively. In the male specimen of Dryotomicus oenophilis, the frons has one median and two large lateral carinae and in the male specimen of Dryotomicus woodrex, the frons has three smaller median tubercles arranged transversely. Phloeotribus puberulus Chapuis and Phloeotribus tuberculatus (Eggers) were monophyletic with the new Dryotomicus species and thus are transferred to this genus. Keys to the Phloeotribini genera and Dryotomicus species are given.

## Introduction

The most diverse and unknown scolytine fauna lies in the tropics. Although a recent monograph of the South American scolytines has been published, approximately another 2500 species remain undiscovered in the Neotropics (Wood 2007). Among these species, are lineages with previously unobserved morphologies, some of which represent undescribed genera. For example, Dole and Cognato (2007) described Akrobothrus ecuadoriensis because of the elytral depression around the scutellum, which is a rare character among scolytines. Similarly, we recently discovered two morphologically interesting species of the Phloeotribini collected from primary wet forests in Guyana and Peru. Although the pseudo-lamellate antennal club places these new species in Phloeotribus Latreille, the unusually long funicle and scape suggests the placement of these species in a different genus. Phloeotribini currently contains two genera: Phloeotribus which is represented by ~100 species distributed in the Holarctic, South America (with highest diversity), and Australia and Aricerus Blandford which is represented by three Australian- New Guinea species (Wood 1986). However, as many as nine previously recognized genera have been synonymized with Phloeotribus and, of these, the Neotropical genera Eulytocerus Blandford and Dryotomicus Wood resemble the recently collected specimens based on previous descriptions (Chapuis 1869; Blandford 1897; Schedl 1962; Wood 1962).

In this study, we assembled specimens of Neotropical, Nearctic, and Australian Phloeotribini and conducted a cladistic analysis, which justified the resurrection of Dryotomicus and the description of two new species.

## Materials and methods

Specimens of one Chramesus (outgroup), one Aricerus and 11 Phloeotribus species, which included all species described as Dryotomicus and Eulytocerus (Wood and Bright 1992), fromthe A.J. Cook Arthropod Research Collection, East Lansing, MI [MSUC], the NationalMuseum of Natural History at the Smithsonian Institute, Washington, D.C. [USNM], Snow Insect collection, Lawrence, KS [SMEC], The Natural History Museum, London [BMNH] and Institut Royal des Sciences Naturelles de Belgique (IRSNB) were examined and scored for 20 morphologically variable characters (Tables 1, 2).

**Table 1.** Characters and their states used in the phylogenetic analysis.

Character 1	Antennal club. 0: segments fused, 1: segments articulated.
Character 2	Antennal club, shape of first segment. 0: not applicable, 1: asymmetrical with a constant width, 2: Asymmetrical, expanded at base (j- shaped), 3: symmetrical chevron-shaped.
Character 3	Second funicular segment. 0: not longer than total length of segments 3-5, 1: longer than total length of segments 3-5.
Character 4	Length of scape. 0: not extending beyond the anterior margin of the pronotum, 1: extending beyond the anterior margin of the pronotum.
Character 5	Area between the antennal insertions and mandibles. 0: less than or equal to length of the mandibles, 1: greater than the length of the mandibles.
Character 6	Male frons with median tubercule(s). 0: absent, 1: one 2: > one.
Character 7	Male frons with lateral carina. 0: absent, 1: present.
Character 8	Male head sulcate from the apex of the eyes to the vertex. 0: absent, 1: present.
Character 9	Declivitous anterior edge of the pronotum. 0: absent, 1: present.
Character 10	Dorsum of the pronotum. 0: with dense, minute, oppressed setae, 1: with scattered longer erect setae.
Character 11	Anterior and lateral margins of the pronotum. 0: without asperities, 1: with asperities.
Character 12	Basal margin of the elytra. 0: crenulate, 1: carinate.
Character 13	Elytral striae. 0: deeply impressed, 1: shallowly impressed.
Character 14	First and second interstriae on elytral declivity. 0: raised above striae, 1: flush with striae.
Character 15	Third, fifth and seventh elytral interstriae on declivity. 0: with tubercules, 1: smooth.
Character 16	Scales on elytral declivity. 0: absent, 1: present.
Character 17	Tubercles on protibia (male). 0:3, 1:5, 2:6, 3:7, 4:8, 5:9.
Character 18	Protibiae. 0: without socketed teeth, 1: with socketed teeth.
Character 19	Metatibae. 0: widest at distal end, 1: widest near the middle.
Character 20	Metatibae. 0: with less than five denticles, 1: with more than five denticles.

Using this data matrix (Table 2), most parsimonious trees (mpts) were reconstructed by a branch and bound search in PAUP* 4.0 b10 PPC using default settings (Swofford 2002). Bootstrap values were determined by performing 500 pseudo-replicates in a heuristic search with simple stepwise addition replicates. Bremer support was calculated with TreeRot v.2 (Sorenson 1999).

**Table 2.** Character states used for the reconstruction of the Phloeotribini phylogeny (Fig. 1). Characters and states are in Table 1.

	Characters																			
	1	2	3	4	5	6	7	8	9	10	11	12	13	14	15	16	17	18	19	20
Aricerus sp.	1	3	0	0	0	0	1	0	0	0	1	1	0	0	0	0	0	0	0	0
Phloeotribus championi	1	2	0	0	0	0	1	0	0	1	0	1	0	0	0	0	5	1	1	1
Phloeotribus sp. Costa Rica	1	1	0	0	0	0	0	0	0	1	1	0	0	0	1	0	?	1	0	1
Phloeotribus sp. Ecuador	1	1	0	0	0	0	0	0	0	1	1	0	0	0	0	0	2	1	1	1
Phloeotribus frontalis	1	2	0	0	0	0	0	0	0	1	1	0	0	0	1	0	1	1	0	1
Phloeotribus liminaris	1	2	0	0	0	0	0	0	0	1	0	0	0	0	1	0	2	1	0	1
Phloeotribus ovatus	1	1	0	0	0	?	0	0	0	1	1	0	0	0	1	0	3	1	0	?
Phloeotribus sp. Panama	1	1	0	1	0	0	1	0	0	1	1	0	0	0	1	0	2	1	0	1
Dryotomicus oenophillis sp. n.	1	2	1	1	1	1	1	1	1	0	0	1	1	0	1	1	2	1	1	1
Dryotomicus woodrex sp. n.	1	2	1	1	1	2	1	1	1	0	0	1	1	1	0	1	2	1	1	1
Phloeotribus puberulus	1	2	1	1	1	?	?	?	1	0	0	1	1	0	0	1	2	1	1	1
Phloeotribus tuberculatus	?	?	?	?	?	?	?	?	1	0	0	1	1	0	1	1	?	1	1	1
Chramesus sp.	0	0	0	0	0	0	1	0	0	1	1	0	0	1	1	0	4	1	0	1

## Results and discussion

**Figure 1.**
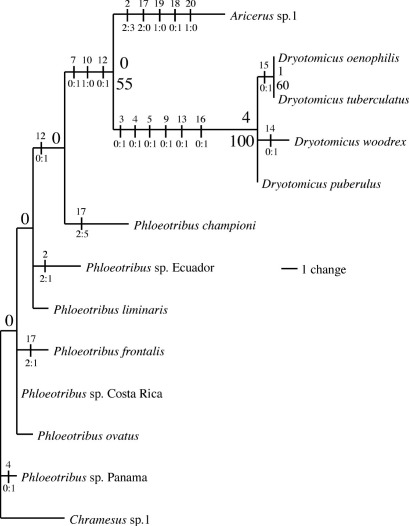
Phylogeny of Phloeotribini species, 1 of 180 most parsimonious trees (tree length=35, RC=0.6429). Numbers above branches are Bremer support and numbers below are bootstrap values (< 50 not shown). Nodes with zero Bremer support were unresolved in a strict consensus of the 180 mpts. Vertical lines represent unambiguous character state changes inferred using accelerated transformations. Numbers above vertical lines are the characters and numbers below are the character state changes.

One hundred-eighty mpts where reconstructed for the 13 taxa. The strict consensus tree of the mpts was mostly unresolved except for the monophyly of Dryotomicus species (Fig. 1). This clade has a high bootstrap value (100), is supported by a relatively high Bremer value (4) and has several diagnostic characters (Fig. 1). The antennal funicle, in which the length of the first two funicular segments equals the last three segments, and the scape, which is twice the length of the funicle, are the most striking features. Antennal morphological variation is taxonomically important because these features diagnose Aricerus as well as species of Phloeotribus (Wood 1982, Wood 1986). Hence, given monophyly and the diagnostic characters, Dryotomicus is removed from synonymy with Phloeotribus and includes four species Dryotomicus puberulus (Chapuis, 1869), Dryotomicus tuberculatus (Eggers, 1943), Dryotomicus oenophilis sp. n., and Dryotomicus woodrex sp. n. Exclusion of Dryotomicus ovatus (Eggers, 1943) and Phloeotribus championi (Blandford, 1897) from this clade confirms their synonymy with Phloeotribus (Wood and Bright 1992). Although the relationship of the Phloeotribus spp. is unresolved, many of the mpts suggest that this genus is potentially paraphyletic. The inclusion of more Phloeotribus spp. and data, especially DNA sequences, in future phylogenetic analyses would help to solidify the relationships among the genera.

The relationship of the four Dryotomicus species is mostly unresolved (Fig. 1). Low support values indicate a potential sister- relationship between Dryotomicus oenophilis and Dryotomicus tuberculatus. However this is a likely spurious result caused by missing character data for Dryotomicus tuberculatus; the only known specimen representing this species is missing its head. However, the distinct morphology of the male frons and elytral declivity distinguish the new species from Dryotomicus tuberculatus (Fig. 1).

## Systematics

### Dryotomicus

Wood genus bona

Dryotomus  Chapuis 1869: 46. Type species: Dryotomus puberulus Chapuis, monobasic, preoccupied by Swainson 1831: 301. Synonymy: Schedl 1962: 487. (References in Wood and Bright 1992: 216)
Dryotomicus Wood 1962: 76. Dryotomus puberulus Chapuis, automatic. Synonymy: Wood 1982: 256. (References in Wood and Bright 1992: 217)

#### Diagnosis.

The asymmetrical first segment of antennal club, socketed teeth on the protibae, and rounded lateral margins of the pronotum distinguish this genus from Aricerus. The usual median tubercle(s) on the male frons, the longer second antennal funicular segment, declivitous anterior edge of the pronotum, the shallowly impressed elytral striae, and elytral declivity with scales and long setae distinguishes Dryotomicus from Phloeotribus.

### Dryotomicus oenophilis sp. n.

urn:lsid:zoobank.org:act:A2174B38-3EBD-4463-AB45-718C449F858D

Figs 2 5 

#### Diagnosis.

Dryotomicus oenophilis is distinguished from the other Dryotomicus species by a large medial tubercle and lateral carina with acute proximal tips on the male frons, interstriae 2 without long uniserial setae, and by raised interstriae of the elytral declivity having tubercles on interstriae 3, 5, and 7 (Fig. 3B).

#### Description.

**Figure 2.**
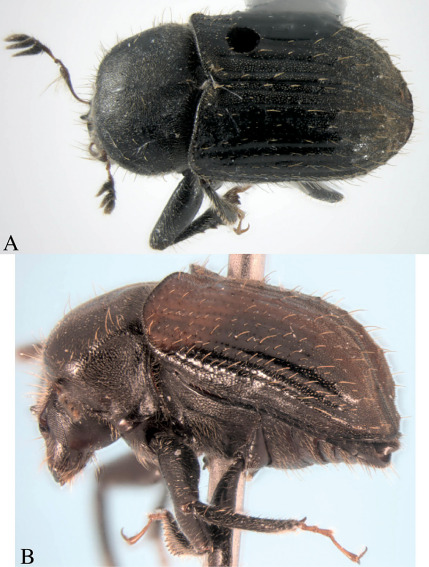
Dryotomicus oenophilis sp. n. male. Habitus, **A** Dorsal **B** Lateral.

*Holotype*, male, total length 4.5 mm (3.8–4.5 mm, n=7), 2× longer than wide, color reddish-black (Fig. 2).

##### Head.

**Figure 3.**
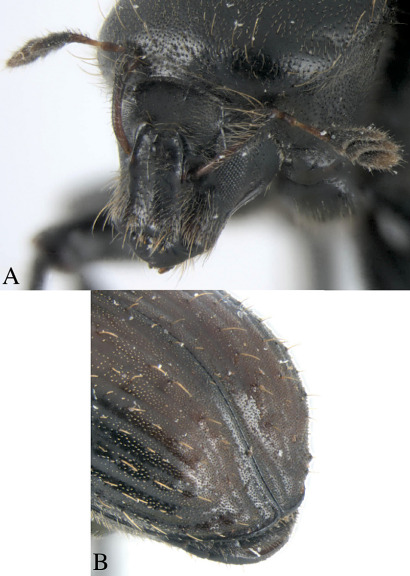
Dryotomicus oenophilis sp. n. male, **A** Frons **B** Elytral declivity.

Frons shagreen with setae as long as or longer than the large median tubercle, longest setae close to epistoma and frontal margins; a large median tubercle between antennal insertions and dorsal margin of eye; lateral carinae from epistoma to dorsal end of eye thicker at antennal insertion and ending acutely (Fig. 3A). Vertex, shagreen with setae approximately as long as or longer than large median tubercle; slightly concave with distinct slightly carinate lateral margins; obtuse median carina from median frontal tubercle to epistoma. Antennae, scape expanded distally and curved proximally beyond the anterior edge of pronotum, funicle five segmented, segments 1 and 2 about equal length and each as long as the combination of segments 3, 4, 5, club pseudo-lamellate, asymmetric, basal segment 1 expanded at base (j- shaped). Eyes oval, ventrally acute (Fig. 3A).

##### Pronotal

width 2.2 mm (1.7–2.2 mm, n=7), 0.64× longer than wide; quadrate flat summit not apparent, densely punctured with appressed minute pubescence and scattered longer setae approximately as long as the funicle concentrated anteriorly and laterally.

##### Elytra

1.2× longer than wide, 2× longer than pronotum, striae on disk impressed, punctures only evident near declivity; striae 4–9 marked by shallow, uniserial punctures; interstriae on disk 2–3× width of striae, confused scales from base to apex on interstriae 1 and 2, interstriae 1 and 3–9 with long uniserial setae approximately as long as the funicle; interstriae 3–9 minutely punctured. (Figs 2, 3B). Elytral declivity with densely placed scales and scattered long setae; striae impressed; interstriae 3, 5, 7 each with 3 tubercles (Fig. 3B).

##### Male genitalia

**Figure 4.**
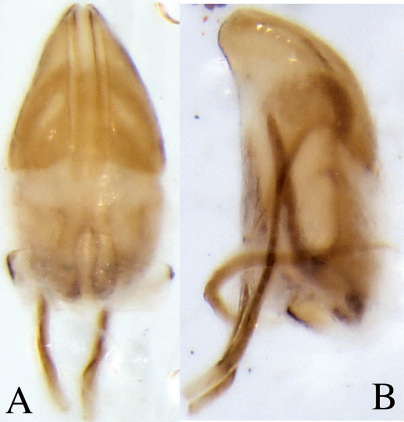
Dryotomicus oenophilis sp. n. male genitalia, **A** Dorsal **B** Lateral. Spiculum gastrale not shown.

Aedeagal body (median lobe) conical, apex acute, lateral margins heavily sclerotized medially on apical half, apophyses (struts) as long as body, attached ventrally; internal sac central area lightly sclerotized, lateral margins heavily sclerotized appearing as ventral apophyses (struts) directed apically , seminal trough at proximal end comprised of two lobes that curve medially (Fig. 4). Tegmen circular, weakly sclerotised on dorsal side. Spiculum gastrale nearly as long as adeagus, crescent-shaped with small knob near the apicalend.

##### Female

**Figure 5.**
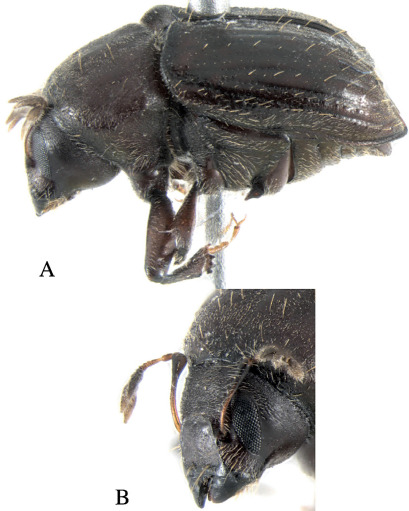
Dryotomicus oenophilis sp. n. female, **A** Lateral habitus **B** Frons.

similar to male in most features, except frons flat to slightly convex, densely punctured, without median tubercles and carinae (Fig. 5A). Strial punctures on elytra more distinct, interstrial tubercles smaller (Fig. 5B).

#### Type material.

Holotype and 6 paratypes (3 males and 3 females) bear two collection data labels, First: “Guyana: Iwokrama Forest, GPS N 04,40.486’, W 58.41.028’, 4–9 March 2007, Cognato, Hulcr, Smith, Dole, McCall Colls”; Second: “Collected with ethanol trap”. The holotype is deposited in the Biodiversity Center at the University of Guyana and 4 paratypes are deposited in the A. J. Cook Arthropod Research Collection, Michigan State University, East Lansing; 2 paratypes are in the U.S. National Museum of Natural History, Washington D.C.

#### Notes.

In Guyana, we collected all specimens from 20 plastic cups filled with 100 ml of 90% ethanol and nailed to trees 1.5 meters above ground.

#### Etymology.

*oeno* (G) = wine, *philis* (G) = lover. The name “wine-lover” signifies the collection of all specimens from ethanol traps.

### Dryotomicus woodrex sp. n.

urn:lsid:zoobank.org:act:C0901E0A-AC6D-4501-B4F4-1E5E975DC0B6

Figs 6– 8 

#### Diagnosis.

Dryotomicus woodrex is distinguished from the other Dryotomicus species by three medial tubercles arranged transversely on a tumescence on the male frons; the interstriae flush with striae on the elytral declivity.

#### Description.

**Figure 6.**
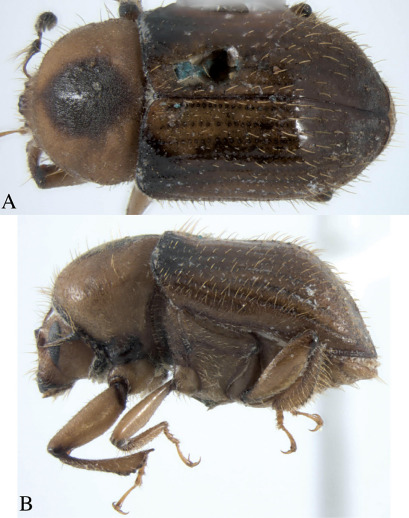
Dryotomicus woodrex sp. n. male. Habitus, **A** Dorsal **B** Lateral.

Holotype, male, total length 4.6 mm, 2× longer than wide, antennae reddish-black, head, legs, thorax, and elytra tannish (perhaps teneral). Pronotum tannish with dark diamond pattern (Fig. 6).

##### Head.

**Figure 7.**
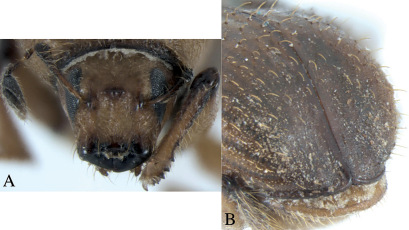
Dryotomicus woodrex sp. n. male, **A** Frons **B** Elytral declivity.

Frons shagreen with setae approximately as long as or longer than funicle; three medial tubercles arranged transversely on a tumescence between antennal insertion and dorsal margin of eye; lateral carinae from epistoma to dorsal end of eye thicker at antennal insertions (Fig. 7A). Vertex shagreen with setae approximately as long or longer than funicle; slightly concave. Antennae, scape expanded distally and curved proximally beyond the anterior edge of pronotum, funicle 5-segmented, segments 1 and 2 about equal length and each as long as segments 3–5 combined, club pseudo-lamellate, asymmetric, segment 1 expanded at base (j- shaped). Eyes oval, ventrally acute (Fig. 3A).

##### Pronotal

width 2.3 mm, 0.65× longer than wide; quadrate disk, summit not distinct, surface densely punctured with appressed pubescence and scattered longer setae approximately as long as funicle concentrated anteriorly and laterally.

##### Elytra

1.2× longer than wide, 2× longer than pronotum, striae on disk not impressed, punctures distinct; interstriae 3–4× width of striae, long uniserial setae approximately as long as funicle arising from granules (Fig. 6). Elytral declivity densely scaled with scattered long setae concentrated along the lateral margin (Fig. 7B).

##### Male genitalia.

**Figure 8.**
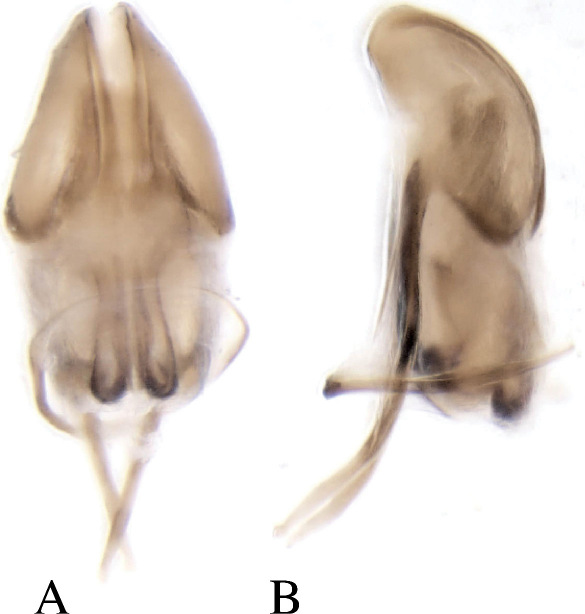
Dryotomicus woodrex sp. n. male genitalia, **A** Dorsal **B** Lateral.

Aedeagal body (median lobe) conical, apex acute, lateral margins heavily sclerotized medially on apical half, apophyses (struts), as long as body, attached ventrally; internal sac central area lightly sclerotized, lateral margins heavily sclerotized appearing as ventral struts directed apically, seminal trough proximal end comprised of two parallel lobes (Fig. 8). Tegmen circular, weakly sclerotised on dorsal side. Spiculum gastrale destroyed by dissection.

##### Female

is unknown.

#### Type material.

Holotype bears the collection data label: “PERU: Dept. Loreto, 1.5km N Teniente Lopez, 4°35.66’S; 76°06.92’W, 22 July 1993, 210–240 m, Richard Leschen #164, ex: flight interception trap”. The holotype is deposited in the Snow Museum, University of Kansas [SMEC].

#### Etymology.

The name “woodrex” honors Dr. Stephen L. Wood’s kingly contribution to the knowledge of scolytine and platypodine taxonomy. It is used as a noun in apposition.

### Dryotomicus puberulus

(Chapuis) comb. n.

Dryotomicus puberulus  (Chapuis) 1869: 46 (Dryotomus). Holotype: female, Cayenne; IRSNB, Brussels. (References in Wood and Bright 1992: 227)

#### Diagnosis.

This species differs from other Dryotomicus spp. by the absence of tubercules from the third, fifth and seventh interstriae and the raised first and second interstriae of elytral declivity.

#### Redescription.

See Wood (2007): 125.

### Dryotomicus tuberculatus

(Eggers) comb. n.

Dryotomicus tuberculatus  (Eggers) 1943: 348 (Dryotomus). Holotype: male ?, Bolivia (Cochabamba); USNM, Washington. (References in Wood and Bright 1992: 235)

#### Diagnosis.

This species differs from other Dryotomicus spp. by the presence of tubercules on the third, fifth and seventh interstriae of the elytral declivity and rugose interstriae of elytral disk and with more than 3 tubercles (Fig. 10)

#### Redescription.

See Wood (2007): 125–126

## Key to Phloeotribini genera

**Table:** 

1.	First segment of antennal club symmetrical chevron-shaped (Fig. 9); protibia without socketed teeth; lateral margin of pronotum marked by asperites; Australia to New Guinea	Aricerus
–	First segment of antennal club asymmetrical; protiba with socketed teeth; lateral margin of pronotum rounded, without asperites	2
2.	Second antennal funicular segment longer than total length of funicular segments 3–5; anterior edge of the pronotum declivitous; elytral declivity with scales and long setae	Dryotomicus
–	Second antennal funicular shorter than total length of funicular segments 3–5; anterior edge of the pronotum flat; elytral declivity with long setae only	Phloeotribus

## Key to Dryotomicus species

**Table:** 

1.	Elytral declivity with tubercules on the third, fifth and seventh interstriae	2
–	Elytral declivity without tubercules on the third, fifth and seventh interstriae	3
2.	Interstriae of elytral disk smooth and with 3 or fewer tubercles	Dryotomicus oenophilis sp.n.
–	Interstriae of elytral disk rugose and with more than 3 tubercles (Fig. 10)	Dryotomicus tuberculatus (Eggers)
3.	First and second interstriae on elytral declivity flush with striae	Dryotomicus woodrex sp. n.
–	First and second interstriae on elytral declivity raised above striae (Fig. 11)	Dryotomicus puberulus (Chapuis)

**Figure 9.**
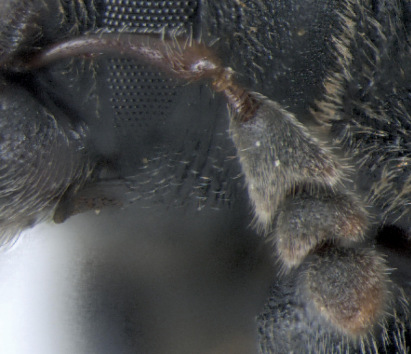
Aricerus sp. antenna.

**Figure 10.**
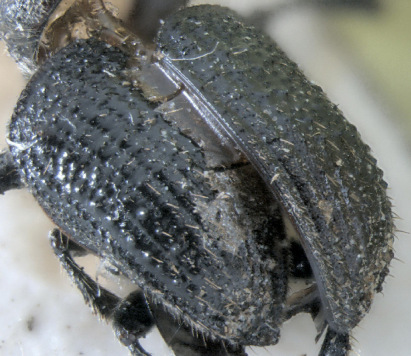
Dryotomicus tuberculatus elytra.

**Figure 11.**
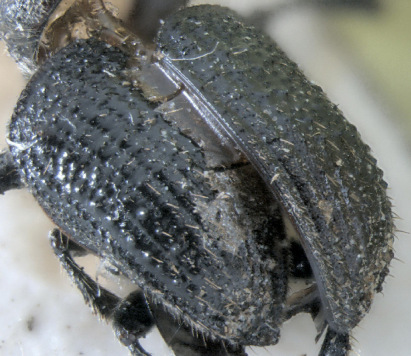
Dryotomicus puberulus elytral declivity.

## Supplementary Material

XML Treatment for Dryotomicus

XML Treatment for Dryotomicus oenophilis

XML Treatment for Dryotomicus woodrex

XML Treatment for Dryotomicus puberulus

XML Treatment for Dryotomicus tuberculatus
